# Medical insurance and healthcare utilization among the middle-aged and elderly in China: evidence from the China health and retirement longitudinal study 2011, 2013 and 2015

**DOI:** 10.1186/s12913-020-05522-w

**Published:** 2020-07-14

**Authors:** Yue Zhou, Haishaerjiang Wushouer, Daniel Vuillermin, Bingyu Ni, Xiaodong Guan, Luwen Shi

**Affiliations:** 1 Department of Pharmacy Administration and Clinical Pharmacy, School of Pharmaceutical Sciences, Peking University, No.38 Xueyuan Road, Haidian District, Beijing, 100191 China; 2 Center for Strategic Studies, Chinese Academy of Engineering, No.2 Bingjiaokou HuTong, Xicheng District, Beijing, 100088 China; 3 School of Medicine, Tsinghua University, No.30 Shuangqing Road, Haidian District, Beijing, 100084 China; 4 School of Health Humanities, Peking University, No.38 Xueyuan Road, Haidian District, Beijing, 100191 China; 5 International Research Center for Medicinal Administration, Peking University, No.38 Xueyuan Road, Haidian District, Beijing, 100191 China; 6 Department of Population Medicine, Harvard Medical School and Harvard Pilgrim Health Care Institute, 133 Brookline Avenue, Boston, MA 02215 USA

**Keywords:** Healthcare utilization, Medical insurance, Aging population

## Abstract

**Background:**

In response to China’s rapidly aging population and increasing healthcare service demands, the Chinese government is developing a universal medical insurance system. This study aimed to assess healthcare utilization patterns and analyze the impacts of medical insurance schemes on healthcare utilization among the middle-aged and elderly in China.

**Methods:**

Data was extracted from the China Health and Retirement Longitudinal Study in 2011, 2013 and 2015. Healthcare utilization was measured by outpatient and inpatient service utilization. Univariate analysis was deployed to examine the impacts of different medical insurance schemes on healthcare utilization. The factors associated with healthcare utilization were estimated using a random-effects logistic regression model.

**Results:**

During the study period, the number of individuals involved was 17,250, 18,195 and 19,842, respectively. The proportion of individuals who received outpatient service was 18.6, 20.7 and 18.7% and those who used inpatient service was 9.6, 13.8 and 14.3%, respectively. We identified that medical insurance was a major protective factor for improving healthcare utilization but different medical insurance schemes exerted various impacts on the middle-aged and the elderly.

**Conclusions:**

Despite the growing population coverage, the Chinese government should make every effort to bridge the gap among people with different medical insurance schemes. Further evaluation is needed to assess whether the expanded medical insurance schemes could protect the middle-aged and elderly households from catastrophic health expenditure.

## Background

Aging populations is becoming a prominent issue facing many countries around the world [1]. In 2017, there were approximately 54 million people aged 45 and above in mainland China, which accounted for up to 40.2% of the population [2]. People have a longer lifespan but also higher morbidity rates due to non-communicable diseases (NCD), such as hypertension, diabetes and cardiovascular disease [3, 4]. In China, an aging population has brought more than 300 million NCD patients across the country which led to increasing healthcare utilization and further straining healthcare services [4–6]. Therefore, providing high-quality healthcare services is of great need [1, 7].

While China’s Opening up and Reform policies have lifted millions of people out of poverty, there is also a growing gulf between the rich and the poor [8, 9]. Low-income groups suffer greater challenges in accessing healthcare services and are more likely to be impoverished due to the health expenditures. This exacerbates the disparities between the rich and the poor in healthcare utilization [10]. In response to these problems, the Chinese government develops a universal medical insurance system, including the Urban Employees Basic Medical Insurance (UEBMI), the New Rural Cooperative Medical Insurance Scheme (NRCMS) and the Urban Residents’ Basic Medical Insurance (URBMI). Specifically, UEBMI, initiated for urban employees and retired employees in 1998, is a mandatory medical insurance scheme. NRCMS, launched in 2003, is a voluntary medical insurance scheme targeting rural populations. In 2007, URBMI was formulated for urban residents without formal employment who could enroll voluntarily. UEBMI is mainly financed by the payroll taxes from beneficiaries. NRCMS and URBMI are financed by the government and premiums, with the government subsidies accounted for majority of the financing [11]. Then, along with the crucial health system reforms in 2009, great strides have been made to consolidate NRCMS and URBMI into the Urban and Rural Resident Medical Insurance. From 2011 to 2015, the premium for NRCMS and URBMI significantly increased and the benefit packages expanded. In general, the list of medical services eligible for reimbursement for NRCMS and URBMI is shorten than that of UEBMI and the reimbursement rate is even lower [11]. The medical insurance schemes mentioned together with private medical insurances (PMI) comprise the national medical insurance system in China [11]. Consequently, near-universal medical insurance coverage has been achieved, from only 85% (1.13 billion) of the Chinese population covered in 2008 to 95% (1.28 billion) in 2011 and 97% (1.33 billion) in 2015 [12–14].

Previous studies found that insurances would lower the barriers for individuals to visit a doctor or have physical examinations. As UEBMI individuals provided the most generous benefit packages, individuals covered by URBMI were more likely to use healthcare services, especially inpatient service [15–18]. However, great strides were made in improving the health system in China and the benefit packages of NRCMS and URBMI were improved. We hypothesized that the promoted medical insurance system could narrow the gaps in healthcare utilization among individuals covered by different medical insurance schemes. Hence, we conducted this study based on a three-year panel data which could track variations in healthcare utilization over time to assess healthcare utilization patterns and analyze the impacts of the medical insurance schemes on healthcare utilization among the middle-aged and elderly in China.

## Methods

### Data source

Data were extracted from the China Health and Retirement Longitudinal Study (CHARLS) (http://charls.pku.edu.cn/) in 2011, 2013 and 2015 [19]. The CHARLS national surveys targeted population aged 45 and above in 150 counties from 28 provinces in China. In our study, the panel data of the 2011 CHARLS Wave 1 (Baseline), the 2013 CHARLS Wave 2 and the 2015 CHARLS Wave 3 were used to describe the patterns of healthcare utilization and evaluate the impacts of medical insurance schemes on healthcare utilization among the middle-aged and elderly in China.

### Study variables

The dependent variable was healthcare utilization measured by outpatient and inpatient service utilization. Outpatient service utilization was defined as visiting but without admission to medical facilities, including general hospitals, specialized hospitals, traditional Chinese medicine hospitals, community healthcare centers, township hospitals, health care posts or village clinic/private clinics in the last 4 weeks for treatment. Inpatient service utilization was defined as being admitted within the last year to a general hospital, specialized hospital, Chinese medicine hospital, community healthcare center, township hospital or health care post [19].

Based on previous literatures, the independent variables included economic status (total household annual expenditure per capita), medical insurance schemes, individual characteristics (gender, age, marital status, educational status, registration place, region), health status and functioning (self-reported health, chronic diseases, depression) and family information (household size) [17, 20, 21]. (Specific information in Table 1).

**Table 1:** Description of variables in 2011, 2013 and 2015 ^a^

Variables	2011 *N* = 17,250	2013 *N* = 18,195	2015 *N* = 19,842
	n (%) ^b^	n (%)	n (%)
**Medical Insurance Schemes**			
NRCMS	12,285 (72.2)	12,879 (72.1)	13,208 (68.8)
UEBMI	2101 (12.4)	2420 (13.6)	2810 (14.6)
URBMI	948 (5.6)	1284 (7.2)	1361 (7.1)
PMI	540 (3.2)	555 (3.1)	185 (1.0)
NONE	1137 (6.7)	726 (4.1)	1634 (8.5)
**Economic Status (Average Total Household Annual Expenditure Per Capita in 2011, 2013 and 2015/US Dollars)**^**c**^			
Quintile 1 (255.1, 371.0, 409.7)	2891 (20.1)	2400 (20.2)	2747 (20.6)
Quintile 2 (520.3, 749.7, 869.5)	2888 (20.1)	2385 (20.1)	2701 (20.3)
Quintile 3 (803.1, 1129.3, 1368.8)	2882 (20.1)	2377 (20.0)	2644 (19.9)
Quintile 4 (1260.0, 1740.9, 2145.3)	2847 (19.8)	2366 (20.0)	2620 (19.7)
Quintile 5 (3318.9, 4396.0, 6052.2)	2866 (19.9)	2360 (19.9)	2606 (19.6)
**Gender**			
Female	8407 (48.7)	8816 (48.5)	9653 (48.7)
Male	8841 (51.3)	9378 (51.5)	10,181 (51.3)
**Age**			
[45,55]	6895 (40.0)	6705 (36.9)	7410 (37.4)
(55,65]	6117 (35.5)	6553 (36.0)	6742 (34.0)
(65, +∞)	4238 (24.6)	4937 (27.1)	5690 (28.7)
**Marital Status**			
Married or Cohabitated	14,990 (87.0)	15,792 (86.8)	17,140 (86.4)
Divorced, Widowed, Never Married, Separated	2248 (13.0)	2396 (13.2)	2696 (13.6)
**Education Status**			
Below Primary School	4765 (27.7)	4825 (26.5)	5021 (25.4)
Primary School	6715 (39.0)	7161 39.4)	8673 (43.8)
Secondary School	3555 (20.6)	3822 (21.0)	3835 (19.4)
High School and Above	2198 (12.8)	2375 (13.1)	2274 (11.5)
**Registration Place**			
Non-rural areas	6985 (40.5)	7348 (40.4)	8032 (40.5)
Rural Areas	10,265 (59.5)	10,843 (59.6)	11,807 (59.5)
**Region**			
Eastern	5990 (34.7)	6290 (34.6)	6957 (35.1)
Central	5654 (32.8)	5954 (32.7)	6438 (32.5)
Western	5603 (32.5)	5940 (32.7)	6442 (32.5)
**Chronic Diseases**^**d**^			
No	5566 (32.5)	6384 (35.4)	7523 (38.2)
Yes	11,573 (67.5)	11,657 (64.6)	12,196 (61.9)
**Self-reported Health**			
Good	3409 (27.7)	4407 (25.6)	4705 (25.4)
Fair	5790 (47.0)	9032 (52.5)	9918 (53. 6)
Poor	3119 (25.3)	3765 (21.9)	3894 (21.0)
**Depression**			
Mild	12,062 (70.4)	13,661 (75.7)	14,467 (73.4)
Moderate	4209 (24.6)	3761 (20.9)	4267 (21.6)
Severe	868 (5.1)	619 (3.4)	985 (5.0)
**Household Size**			
1–2	6274 (36.4)	6424 (35.3)	7972 (40.2)
3–4	5874 (34.1)	6349 (34.9)	9378 (47.3)
≥ 5	5102 (29.6)	5418 (29.8)	2489 (12.6)
**Outpatient Service Utilization**	3117 (18.6)	3653 (20.7)	3615 (18.7)
**Inpatient Service Utilization**	1631 (9.6)	2486 (13.8)	2813 (14.3)

*NRCMS* New Rural Cooperative Medical Insurance Scheme, *UEBMI* Urban Employees Basic Medical Insurance, *URBMI* Urban Residents’ Basic Medical Insurance, *PMI* Private Medical Insurance

^a^ Numbers across the subgroups of some certain characteristics did not add up to the total because of some missing values

^b^ “n” means the number of individuals in each group and “%” means the proportion of individuals in each group

^c^ Average total household annual expenditure per capita in 2011, 2013 and 2015 was 1228.2 US dollars, 1670.3 US dollars and 2138.9 US dollars, respectively

^d^ Chronic diseases included hypertension, dyslipidemia, diabetes or high blood sugar, cancer or malignant tumor (excluding minor skin cancers), chronic lung diseases, such as chronic bronchitis, emphysema (excluding tumors, or cancer), liver disease (except fatty liver, tumors, and cancer), heart attack, stroke, kidney disease (except for tumor or cancer), stomach or other digestive disease (except for tumor or cancer), emotional, nervous, or psychiatric problems, memory-related disease, arthritis or rheumatism and asthma. In the surveys, people were asked whether they had ever been diagnosed with one of the listed chronic diseases and their responses were recorded

### Statistical analysis

According to CHARLS website (http://charls.pku.edu.cn/index/en.html), new respondents will be included to guarantee the sample representativeness for some respondents may be lost or die. To solve this problem, we treated the data as unbalanced panel data.

### Descriptive and univariate analysis

Descriptive analysis was used to summarize the characteristics of the individuals by expressing the results as the absolute frequencies and percentage. Consumer Price Index was used to adjust for inflation [22]. Total household annual expenditure per capita was converted into United States (US) dollars uniformly based on exchange rates in December 2011 (1 US dollar = 6.3281 Yuan) to make the results more comparable to other published studies [23]. Then univariate analysis was used to examine the impacts of different factors on healthcare utilization by the Chi-squared test.

### Logistic regression analysis

Random-effects logistic model for panel data was used to explore factors associated with healthcare utilization among the middle-aged and elderly in China. The model in our study could be defined by:

$$ Logit\left(\mathit{\Pr}\left({y}_{it}=1\right)\right)=\alpha +\sum \limits_{k=1}^k{\beta}_k{x}_{kit}+{\mu}_{it} $$

*k =* 1, 2, 3 …, N; *t* = 2011, 2013, 2015.

Where *y*_*it*_ is whether the individual *i*_*th*_ used healthcare services for the *t*_*th*_ time period, *α* refers to intercept, *β*_*k*_ refers to coefficient vector which describe the effects of variables (If *β*_*k*_ is over zero, the variable has a positive impact on *y*_*it*_ compared with the reference group and vice versa), *x*_*kit*_ denotes the value of the *k*_*th*_ variable on the *i*_*th*_ people for the *t*_*th*_ time period and *μ*_*it*_ is the random error term.

Stata® version 14.2 was used to perform the data analysis. *P* < 0.05 was considered statistically significant.

## Results

### Sample individual characteristics

Table 1 showed the individual characteristics of the sample population. After excluding invalid data, the number of individuals involved in the three surveys was 17,250, 18,195 and 19,842, respectively. NRCMS covered most of the sampled individuals (72.2, 72.1 and 68.8%), followed by UEBMI (12.4, 13.6 and 14.6%). The proportion of individuals covered by no medical insurance scheme was 6.7, 4.1 and 8.5%, respectively. The average total household annual expenditure per capita was 1254.4 US dollars, 1795.8 US dollars and 2378.4 US dollars, respectively. Female individuals accounted for 48.7, 48.5 and 48.7%, respectively. The proportion of individuals over 65 years old was 24.6, 27.1 and 28.7%. Individuals with chronic diseases accounted for 67.5, 64.6 and 61.9%. The proportion of individuals with poor self-reported health also showed a decreasing trend, which was 25.3, 21.9 and 21.0%.

### Outpatient and inpatient service utilization

As displayed in Table 1, in each of the three surveys of study, the proportion of individuals who received outpatient service was 18.6, 20.7 and 18.7% and who received inpatient service was 9.6, 13.8 and 14.3%, respectively. Univariate analysis showed that significant differences existed between different medical insurance schemes (*p* < 0.05) as well as different economic status (*p* < 0.05) (Table 2).

**Table 2:** Healthcare utilization patterns and univariate analysis in the middle-aged and elderly in 2011, 2013 and 2015

Variables	Outpatient Service Utilization						Inpatient Service Utilization					
	2011 *N* = 17,250		2013 *N* = 18,195		2015 *N* = 19,842		2011 *N* = 17,250		2013 *N* = 18,195		2015 *N* = 19,842	
	n (%) ^a^	*p*	n (%)	*p*	n (%)	*p*	n (%)	*p*	n (%)	*p*	n (%)	*p*
**Medical Insurance Schemes**		**0.000**		**0.000**		**0.000**		**0.000**		**0.000**		**0.000**
NRCMS	2371 (19.6)		2706 (21.4)		2528 (19.4)		1129 (9.2)		1753 (13.6)		1830 (13.9)	
UEBMI	328 (16.0)		476 (20.2)		502 (18.3)		277 (13.2)		418 (17.3)		485 (17.3)	
URBMI	163 (17.5)		236 (18.8)		237 (17.7)		101 (10.7)		179 (14.0)		220 (16.2)	
PMI	100 (18.7)		93 (17.1)		36 (19.8)		59 (10.9)		59 (10.7)		27 (14.7)	
NONE	150 (13.4)		114 (16.0)		227 (14.1)		59 (5.2)		56 (7.7)		195 (12.0)	
**Economic Status**		**0.011**		**0.001**		**0.004**		**0.000**		**0.000**		**0.000**
Quintile 1 (Poorest)	473 (16.6)		433 (18.2)		440 (16.2)		176 (6.1)		230 (9.6)		278 (10.1)	
Quintile 2	565 (19.8)		482 (20.5)		500 (18.8)		233 (8.1)		279 (11.7)		283 (10.5)	
Quintile 3	528 (18.6)		468 (20.1)		483 (18.6)		275 (9.6)		314 (13.2)		365 (13.8)	
Quintile 4	552 (19.7)		470 (20.2)		471 (18.3)		318 (11.2)		349 (14.8)		421 (16.1)	
Quintile 5	534 (19.1)		532 (23.2)		512 (20.3)		378 (13.2)		407 (17.3)		471 (18.2)	
**Gender**		**0.000**		**0.000**		**0.000**		0.706		0.736		**0.025**
Female	1325 (16.2)		1558 (18.1)		1561 (16.5)		803 (9.6)		1214 (13.9)		1315 (13.7)	
Male	1792 (20.8)		2095 (23.1)		2054 (20.7)		828 (9.5)		1272 (13.7)		1498 (14.9)	
**Age**		**0.000**		**0.000**		**0.000**		**0.000**		**0.000**		**0.000**
[45,55]	1118 (16.7)		1242 (19.1)		1259 (17.4)		459 (6.7)		661 (10.0)		724 (9.9)	
[55,65]	1140 (19.1)		1290 (20.2)		1225 (18.6)		593 (9.8)		900 (13.8)		937 (14.0)	
(65, +∞)	859 (20.9)		1121 (23.4)		1131 (20.4)		579 (13.9)		925 (19.1)		1152 (20.5)	
**Marital Status**		**0.010**		**0.000**		**0.017**		0.108		**0.000**		**0.000**
Married or Cohabitated	2667 (18.3)		3102 (20.2)		3081 (18.4)		1398 (9.4)		2093 (13.4)		2305 (13.6)	
Divorced, Widowed, Never Married, Separated	450 (20.6)		551 (23.9)		534 (20.4)		233 (10.5)		393 (16.7)		508 (19.1)	
**Education Status**		**0.000**		**0.000**		**0.006**		**0.043**		**0.000**		**0.000**
Below Primary School	979 (21.0)		1057 (22.5)		999 (20.3)		482 (10.2)		693 (14.5)		824 (16.5)	
Primary School	1252 (19.1)		1475 (21.1)		1564 (18.4)		654 (9.8)		1058 (14.9)		1208 (14.0)	
Secondary School	560 (16.2)		713 (19.3)		658 (17.6)		314 (8.9)		433 (11.5)		502 (13.2)	
High School and Above	325 (15.4)		408 (17.9)		390 (17.9)		180 (8.3)		301 (12.9)		275 (12.3)	
**Registration Place**		**0.000**		**0.025**		**0.003**		**0.002**		**0.000**		**0.010**
Non-rural areas	1138 (16.9)		1395 (19.8)		1368 (17.6)		715 (10.4)		1081 (15.0)		1194 (15.1)	
Rural areas	1979 (19.7)		2258 (21.2)		2247 (19.3)		916 (9.0)		1405 (13.0)		1619 (13.8)	
**Region**		0.085		**0.000**		**0.000**		**0.000**		**0.000**		**0.000**
Eastern	1032 (17.7)		1154 (18.8)		1178 (17.4)		439 (7.4)		693 (11.2)		827 (12.0)	
Central	1039 (18.8)		1142 (19.8)		1137 (18.0)		535 (9.5)		806 (13.7)		891 (14.0)	
Western	1046 (19.2)		1357 (23.6)		1300 (20.7)		657 (11.9)		986 (16.8)		1095 (17.2)	
**Household Size**		**0.000**		**0.000**		0.758		**0.000**		**0.000**		**0.000**
1–2	1132 (18.6)		1244 (20.0)		1452 (18.7)		660 (10.7)		981 (15.5)		1237 (15.7)	
3–4	977 (17.1)		1244 (20.2)		1695 (18.5)		484 (8.3)		766 (12.2)		1276 (13.7)	
≥5	1008 (20.2)		1165 (22.0)		468 (19.2)		487 (9.6)		739 (13.8)		300 (12.1)	
**Chronic Diseases**^b^		**0.000**		**0.000**		**0.000**		**0.000**		**0.000**		**0.000**
No	545 (10.0)		901 (14.4)		1015 (13.7)		246 (4.5)		532 (8.4)		718 (9.6)	
Yes	2571 (22.7)		2736 (24.0)		2597 (21.7)		1384 (12.0)		1947 (16.8)		2093 (17.2)	
**Self-reported Health**		**0.000**		**0.000**		**0.000**		**0.000**		**0.000**		**0.000**
Good	277 (8.2)		483 (11.1)		450 (9.7)		141 (4.2)		305 (7.0)		308 (6.6)	
Fair	952 (16.8)		1741 (19.7)		1742 (17.8)		478 (8.3)		1052 (11.7)		1187 (12.0)	
Poor	938 (30.9)		1264 (34.6)		1212 (32.1)		579 (18.6)		975 (26.0)		1106 (28.5)	
**Depression**		**0.000**		**0.000**		**0.000**		**0.000**		**0.000**		**0.000**
Mild	1779 (15.1)		2388 (17.9)		2271 (16.0)		978 (8.2)		1620 (12.0)		1742 (12.1)	
Moderate	1074 (25.9)		1028 (27.9)		1014 (24.3)		510 (12.1)		703 (18.7)		808 (19.0)	
Severe	263 (31.0)		221 (37.0)		327 (33.9)		142 (16.4)		156 (25.3)		261 (26.5)	

*NRCMS* New Rural Cooperative Medical Insurance Scheme, *UEBMI* Urban Employees Basic Medical Insurance, *URBMI* Urban Residents’ Basic Medical Insurance, *PMI* Private Medical Insurance

^a^ “n” means the number of individuals in each group who used healthcare service and “%” means the proportion of individuals in each group who used healthcare service

^b^ Chronic diseases included hypertension, dyslipidemia, diabetes or high blood sugar, cancer or malignant tumor (excluding minor skin cancers), chronic lung diseases, such as chronic bronchitis, emphysema (excluding tumors, or cancer), liver disease (except fatty liver, tumors, and cancer), heart attack, stroke, kidney disease (except for tumor or cancer), stomach or other digestive disease (except for tumor or cancer), emotional, nervous, or psychiatric problems, memory-related disease, arthritis or rheumatism and asthma. In the surveys, people were asked whether they had ever been diagnosed with one of the listed chronic diseases and their responses were recorded

### Impacts of medical insurance schemes on healthcare utilization

Table 3 demonstrated the impacts of medical insurance schemes on healthcare utilization. Specifically, individuals covered by medical insurance schemes were more likely to receive both outpatient and inpatient services than individuals with no medical insurance schemes (*p* < 0.05). Compared with individuals covered by NRCMS, individuals covered by URBMI were less likely to receive outpatient service (*p* < 0.05), while individuals covered by UEBMI were more likely to receive inpatient service (*p* < 0.05). There was a positive correlation between economic status and healthcare utilization, that is, the richer the individuals were, the more healthcare services were likely to be receive (*p* < 0.05). What’s more, individuals older than 65 years old, living in a household of more than 2 people, with chronic diseases, fair-to-poor self-reported health, or with moderate to high depression were more likely to receive both outpatient and inpatient services (*p* < 0.05). Besides, individuals with higher education status or locating in rural areas were more likely to receive outpatient service. The situation was the opposite for inpatient service utilization even the statistical results were not significant.

**Table 3:** Determinants of healthcare utilization in the middle-aged and elderly in 2011, 2013 and 2015

Variables	Reference Group	Outpatient Service Utilization			Inpatient Service Utilization		
		Coefficient	SE	*p*	Coefficient	SE	*p*
**Gender**	Female						
Male		**0.239**	**0.037**	**0.000**	− 0.062	0.045	0.168
**Age**	[45,55]						
(55,65]		−0.008	0.041	0.837	**0.345**	**0.052**	**0.000**
(65, +∞)		**0.169**	**0.049**	**0.001**	**0.739**	**0.060**	**0.000**
**Marital Status**	Married or Cohabitated						
Divorced, Widowed, Never Married, Separated		−0.030	0.056	0.590	0.011	0.065	0.869
**Education Status**	Below Primary School						
Primary School		0.067	0.046	0.143	0.005	0.055	0.922
Secondary School		0.094	0.057	0.101	−0.095	0.070	0.176
High School and Above		**0.148**	**0.071**	**0.036**	**−0.260**	**0.087**	**0.003**
**Registration Place**	Non-rural areas						
Rural areas		0.053	0.041	0.192	−0.087	0.050	0.078
**Region**	Eastern						
Central		−0.072	0.043	0.094	**0.182**	**0.054**	**0.001**
Western		0.039	0.043	0.356	**0.398**	**0.053**	**0.000**
**Household Size**	1–2						
3–4		**0.142**	**0.040**	**0.000**	**0.126**	**0.048**	**0.008**
≥5		**0.249**	**0.046**	**0.000**	**0.139**	**0.056**	**0.013**
**Chronic Diseases**^**a**^	No						
Yes		**0.467**	**0.040**	**0.000**	**0.403**	**0.049**	**0.000**
**Self-Reported Health**	Good						
Fair		**0.630**	**0.046**	**0.000**	**0.625**	**0.059**	**0.000**
Poor		**1.364**	**0.055**	**0.000**	**1.584**	**0.067**	**0.000**
**Depression**	Low						
Moderate		**0.315**	**0.039**	**0.000**	**0.254**	**0.047**	**0.000**
High		**0.480**	**0.072**	**0.000**	**0.367**	**0.084**	**0.000**
**Economic Status**	Quintile 1 (Poorest)						
Quintile 2		**0.243**	**0.052**	**0.000**	**0.258**	**0.068**	**0.000**
Quintile 3		**0.283**	**0.053**	**0.000**	**0.609**	**0.067**	**0.000**
Quintile 4		**0.317**	**0.054**	**0.000**	**0.796**	**0.067**	**0.000**
Quintile 5		**0.494**	**0.056**	**0.000**	**1.058**	**0.070**	**0.000**
**Medical Insurance Schemes**	NRCMS						
UEBMI		0.009	0.060	0.882	**0.340**	**0.070**	**0.000**
URBMI		**−0.172**	**0.072**	**0.016**	0.136	0.082	0.095
PMI		0.024	0.109	0.827	0.226	0.131	0.083
NONE		**−0.422**	**0.077**	**0.000**	**−0.545**	**0.100**	**0.000**

McKelvey & Zavoina’s R^2^ of the model used in our study were 0.1027 (outpatient service utilization) and 0.1493(inpatient service utilization)

*NRCMS* New Rural Cooperative Medical Insurance Scheme, *UEBMI* Urban Employees Basic Medical Insurance, *URBMI* Urban Residents’ Basic Medical Insurance, *PMI* Private Medical Insurance

^a^ Chronic diseases included hypertension, dyslipidemia, diabetes or high blood sugar, cancer or malignant tumor (excluding minor skin cancers), chronic lung diseases, such as chronic bronchitis, emphysema (excluding tumors, or cancer), liver disease (except fatty liver, tumors, and cancer), heart attack, stroke, kidney disease (except for tumor or cancer), stomach or other digestive disease (except for tumor or cancer), emotional, nervous, or psychiatric problems, memory-related disease, arthritis or rheumatism and asthma. In the surveys, people were asked whether they had ever been diagnosed with one of the listed chronic diseases and their responses were recorded

## Discussion

We observed that outpatient service utilization increased in 2013 and then decreased in 2015, while inpatient service utilization increased over time with distinct changes. This indicated that the demands of inpatient care may still not be met. We identified that medical insurance schemes would improve healthcare utilization but different medical insurance schemes exerted various impacts on the middle-aged and elderly.

In our study, more than 60% of the individuals suffered from NCD and about 75% of the individuals did not claim good self-reported health, which indicated the grim reality that there were significant healthcare service demands [24, 25]. It was witnessed a decreasing trend in chronic disease conditions as well as poor self-reported health in contrast to the increasing healthcare service utilization. This may be partly due to improvement for chronic disease prevention and management which allowed the patients to better access the healthcare service [26]. We also found that during the study period, the average utilization rate of outpatient service and inpatient service for the middle-aged and elderly were among 19.3 and 12.7%, respectively, which was consistent with Nation Health Services Survey in China conducted in 2013 (outpatient: 13.7% ~ 26.4%; inpatient: 7.3% ~ 19.9%) [27]. Additionally, we found that the proportion of individuals covered by medical insurance increased in 2013 and then decreased in 2015. Above all, the majority of respondents were members of the NRCMS voluntary scheme. The decrease in medical insurance coverage in our study may be due to the lack of health literacy for the insured to reapply for their insurance schemes. Besides, individuals may choose not to renew membership because they were healthy enough or they failed to receive high-quality services as needed.

We found that individuals with a higher economic status tended to receive more outpatient service and inpatient service, which aligned with the study conducted in Gansu and Zhejiang provinces [15]. Economic status was fundamental to healthcare utilization of individuals and was one of the essential inequity elements favoring the better-off [28, 29]. Despite rapid economic growth following China’s economic reforms in the late 1970s, the gap between the rich and the poor has also increased dramatically [9]. Although the expanded medical insurance could partly alleviate the burden of healthcare service, the impacts were fairly limited. The significant regional disparities in both healthcare resources and quality may impede the healthcare accessibility and affordability of individuals [30].

In our study, individuals covered by medical insurance schemes tended to receive more healthcare services. As a social security system, medical insurance schemes were established to guarantee healthcare utilization as needed and spread the risk of medical expenditures through the co-payment mechanism [31, 32]. Thus, this finding demonstrated that the expanded medical insurance schemes in China may stimulate the healthcare-seeking behaviors of the insured and unleash healthcare demands of the disadvantaged population in general, which was broadly consistent with the existing literature [33–35]. However, improved access to healthcare services may also vary among individuals with different medical insurance schemes because of the various benefit packages and the different financing schemes [36]. For instance, URBMI did not cover outpatient service so the insured had to pay for outpatient by themselves, while individuals covered by NRCMI could reimburse partial outpatient expenses. This could partly explain our finding that compared with NRCMS, URBMI decreased the likelihood of using outpatient service [11]. We also observed that individuals covered by UEBMI were more likely to receive inpatient service than their counterparts covered by NRCMS. Comparing to NRCMS, UEBMI had more generous benefit package and a higher reimbursement rate [11]. This may motivate the insured to seek healthcare services when sick with chronic or acute conditions. Lastly, the target of UEBMI was employees and retirees in urban areas, who generally had better economic conditions than people in rural areas. This might induce the insured to take advantage of healthcare services and may result in overconsumption of healthcare services [11].

However, there are several limitations subject to this study. All information about healthcare utilization, health status as well as economic status was self-reported and therefore inevitably demonstrated memory bias. Besides, the study population was not the same in three surveys (unbalanced panel data), and the benefit packages of different medical insurance schemes were expanded during the study period. This may affect the impacts of medical insurance schemes on healthcare utilization. Moreover, healthcare utilization was measured by outpatient and inpatient visits. The perspective of healthcare utilization assessment was incomprehensive to some extent. Further study should also take indicators such as the intensity of healthcare services into consideration.

## Conclusions

Our findings indicated that increased medical insurance coverage can increase the likelihood of healthcare utilization, especially the inpatient service utilization. Variation of the impacts can be identified among different medical insurance schemes. Further evaluation is needed regarding whether the expanded medical insurance schemes can protect the middle-aged and elderly households from catastrophic health expenditure.

## Data Availability

The datasets supporting the conclusions of this article are available in China Health and Retirement Longitudinal Study (http://charls.pku.edu.cn/). Researchers can log on the website to register by signing the agreement and provide real personal information for reviewing. After completing the registration, researchers could apply for publicly available datasets respectively, and the application will be reviewed and approved in 3 working days. If researchers have any questions or suggestions, they can contact CHARLS by email charls_info@pku.edu.cn or by phone 86–400–610-1866 or 86-(0)10–62767425.

## Abbreviations

CHARLS: China Health and Retirement Longitudinal Study
NCD: Non-Communicable Diseases
NRCMS: New Rural Cooperative Medical Insurance Scheme
PMI: Private Medical Insurance
UEBMI: Urban Employees Basic Medical Insurance
URBMI: Urban Residents’ Basic Medical Insurance
